# Transient Osteoporosis of the Hip: A Case Report

**DOI:** 10.5704/MOJ.1703.012

**Published:** 2017-03

**Authors:** K Pande, TT Aung, JF Leong, I Bickle

**Affiliations:** Department of Orthopaedics, Raja Isteri Pengiran Anak Saleha Hospital, Bandar Seri Begawan, Brunei Darussalam; * Department of Radiology, Raja Isteri Pengiran Anak Saleha Hospital, Bandar Seri Begawan, Brunei Darussalam

**Keywords:** transient osteoporosis of the hip, MRI, bisphosphonate, alendronate sodium

## Abstract

Transient osteoporosis of the hip (TOH) is a benign, selflimiting condition characterised by acute onset groin pain in adults. Early diagnosis is important to differentiate it from progressive conditions such as osteonecrosis. We report on a middle-aged male who presented with right groin pain without any prior trauma. The diagnosis of transient osteoporosis of hip was confirmed by Magnetic Resonance Imaging (MRI) and he was successfully treated with a course of Alendronate sodium, anti-inflammatory analgesics and a period of non-weight bearing ambulation.

## Introduction

Transient osteoporosis of the hip (TOH) is an idiopathic, selflimiting condition observed mostly in men in the fourth or fifth decade of life and women in the third trimester of pregnancy or the immediate postpartum period^1^, ^2^. The diagnosis can be suspected on the history and examination, but MRI scan is essential for confirmation and to differentiate it from other serious conditions. We report a case of a patient with transient osteoporosis of the hip treated with conservative measures and daily alendronate therapy with complete resolution of symptoms and MRI changes.

## Case Report

A 43 years old Asian gentleman presented with acute onset of right thigh pain, over the anterolateral aspect of two weeks duration. There were no significant findings on clinical examination and he was referred for physiotherapy.

**Fig. 1:**
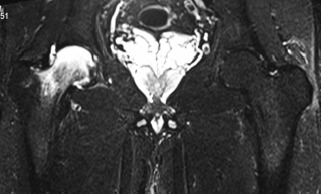
Coronal Short TI Inversion Recovery (STIR) of the hips: Diffuse high T2 signal throughout the right femoral head and neck. No cortical irregularity or collapse.

On review one month later, the pain was persistent and now localized to the groin with local tenderness and terminal restriction of movements. Blood tests including inflammatory markers, serum uric acid and serum rheumatoid factor were negative. An MRI scan of the pelvis was requested to obtain further detail and it confirmed typical changes of transient osteoporosis of the right hip (Fig. 1). The patient was advised on non-weight bearing ambulation with hip range of movements exercises and prescribed anti-inflammatory medications, Alendronate sodium 10mg per day with calcium and vitamin D supplements.

**Fig. 2:**
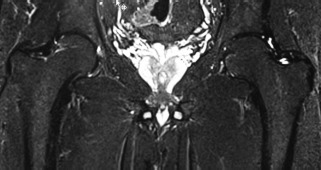
Coronal Short TI Inversion Recovery (STIR) of the hips: Normal marrow signal in the right femoral neck and head. The diffuse high T2 signal on the prior study (Figure 1) has completely resolved. Both images acquired on a Siemens Verio 3T (Erlangen, Germany).

At follow up seven weeks later, the patient had complete relief of pain with full painless range of movements. At further review five months after diagnosis and treatment the patient was mobilizing with full weight bearing. He had a full and painless range of movements at the right hip. Repeat MRI scan confirmed complete resolution of changes (Fig. 2). With the above response, Alendronate sodium was discontinued.

At review seven months after diagnosis, the patient was pain free with full range of movement at the affected hip and had resumed normal activities. He was discharged from outpatient follow up care.

## Discussion

Transient osteoporosis is a rare clinical syndrome that was first described by Curtiss and Kincaid in 1959. TOH typically involves the middle-aged population and is more frequent in men than women. In women, it is more common during the third trimester of pregnancy. It is idiopathic with no history of trauma^1^. The cause and pathogenesis of this condition is unclear. Various local and systemic theories for its causation are proposed. These include pathology of the proximal nerve roots, regional accelerated phenomenon for bone re-modelling and systemic bone loss^1^.

This uncommon condition has been reported in the literature only in the form of case reports and small case series^1^-^4^. In all cases, including the present case, there is spontaneous groin pain of sudden onset with difficulty in weight bearing with only minimal restriction of hip movements. Blood tests are essentially normal, but should be performed to rule out possibility of infection or metastatic disease^1^. Lack of specific symptoms and rarity of the condition often lead to delay in diagnosis. Magnetic resonance imaging is the most sensitive imaging modality for early detection of this condition and to exclude other entities, particularly osteonecrosis and occasionally inflammatory arthritis, neoplasm and osteomyelitis. It has also been used for monitoring of disease progression^5^. Typically, T1 weighted images reveal low signal intensity while inversion recovery sequences images reveal matching high signal intensity in bone marrow with ill-defined edges. MRI returns to normal values when clinical symptoms have disappeared as was seen in the present case^1^,^2^,^5^.

Early differentiation of TOH and osteonecrosis is crucial as the natural history, treatment and outcome of the two conditions are different. TOH is known to have complete clinical and radiological recovery while untreated osteonecrosis of the femoral head leads to progressive damage and arthritis of the hip^1^. Treatment for TOH is mostly conservative which consists of; observation, assurance and symptomatic relief. Now various pharmaceutical agents have been used to treat this condition^1^. The successful use of oral alendronate and other bisphosphontaes has been reported in the literature. The mechanism of their action in TOH is unknown, but is possibly related to their anti-inflammatory properties^2^,^4^.

In conclusion, a high level of suspicion is needed for early diagnosis of TOH. Typical MRI findings are diagnostic and unnecessary invasive investigation and intervention can be avoided. The treatment of this self-limiting condition is symptomatic and bisphosphonates including alendronate sodium are useful in its management.
